# Designing the Future of Children’s Mental Health Services

**DOI:** 10.1007/s10488-020-01038-x

**Published:** 2020-04-06

**Authors:** Aaron R. Lyon, Alex R. Dopp, Stephanie K. Brewer, Julie A. Kientz, Sean A. Munson

**Affiliations:** 1 Department of Psychiatry and Behavioral Sciences, University of Washington, 6200 NE 74th Street, Suite 100, Seattle, WA 98115 USA; 2 RAND Corporation, 1776 Main St, Santa Monica, CA 90401 USA; 3 Department of Human Centered Design and Engineering, University of Washington, 428 Sieg Hall, Seattle, WA 98195 USA

**Keywords:** Human-centered design, User-centered design, Youth mental health, Implementation, Evidence-based practice

## Abstract

Advancements in evidence-based psychosocial interventions, digital technologies, and implementation strategies (i.e., health services research products) for youth mental health services have yet to yield significant improvement in public health outcomes. Achieving such impact will require that these research products are easy to use, useful, and contextually appropriate. This paper describes how human-centered design (HCD), an approach that aligns product development with the needs of the people and settings that use those products, can be leveraged to improve youth mental health services. We articulate how HCD can advance accessibility, effectiveness, and equity, with specific consideration of unique aspects of youth mental health services.

## Introduction

In the United States, one out of every five youth suffer from a diagnosable mental health problem (Centers for Disease Control 2013). During the past four decades, considerable effort has been devoted to testing the effectiveness of health services research products (HSRPs) such as evidence-based psychosocial interventions (EBPIs), digital mental health technologies, and implementation strategies to address child and adolescent mental health needs (see Table 1). These advances offer potential for widespread impact; however, the ability of HSRPs to shift public health outcomes has remained limited (Cabassa 2016; Hollis et al. 2015; Proctor et al. 2009). Although implementation success is determined by a wide range of multilevel barriers and facilitators (including those at the system, organizational, individual, and intervention levels) (Damschroder et al. 2009), a potential mismatch between HSRPs and the real-world needs of the providers, clients, and service settings in which children and adolescents receive care is a critical and under-addressed component of the implementation process (Cabassa 2016; Chambers and Norton 2016). Much of this has been driven by a traditional focus on internal validity in research that often leads interventions, digital technologies, and implementation strategies to be *over-designed* for performance in research and *under-designed* for addressing the needs and constraints of routine service contexts (Lyon and Koerner 2016; Mohr et al. 2017a).

**Table 1:** Health services research products (EBPIs, digital technologies, implementation strategies) with definitions and examples

Health services research product (HSRP)	Definition	Examples
Evidence-based psychosocial intervention (EBPI)	Interpersonal or informational activities, techniques, or strategies that target biological, behavioral, cognitive, emotional, interpersonal, social, or environmental factors with the aim of reducing symptoms of these disorders and improving functioning or well-being (Institute of Medicine 2015)	Parent training protocols Cognitive behavioral therapy Applied behavior analysis
Digital technology	A broad range of technologies to support users (most typically clinicians or clients) in changing behaviors and cognitions related to mental health and wellness	Devices and wearables Clinical decision support tools Digital therapeutics Mobile health apps
Implementation strategy	Methods or techniques used to enhance the adoption, implementation, and sustainment of a clinical program or practice (Proctor et al. 2013)	Initial training meetings Post-training consultation Leadership training for implementation Clinician motivation enhancement

## Evidence-Based Psychosocial Interventions (EBPIs)

Hundreds of EBPI protocols have been developed (Chorpita et al. 2011), yet usual care settings for youth mental health services are characterized by poor uptake and sustainment of EBPIs and inconsistent quality and effectiveness of care (Hoagwood et al. 2001; Shelton et al. 2018). With increasing urgency, mental health providers are being called upon to adopt EBPIs and sustain their use (Aarons et al. 2011). Unfortunately, most efforts to adopt and sustain EBPIs fail, due to a persistent disconnect between the interventions—which are often developed and tested in non-usual care research settings such as academic medical centers or university clinics—and the real-world requirements and constraints of usual care settings (Cabassa 2016; Lyon and Koerner 2016). Common reasons for an implementation effort to fail include lack of financial resources, lack of ongoing external implementation support following adoption, difficulty attracting and retaining well-qualified staff, perceived lack of fit with provider/organization values, and perceived difficulty of EBPI implementation (Massatti et al. 2008). In particular, a lack of fit between an EBPI and its intended clients, providers, and work setting can be fatal to ongoing implementation success (Rodriguez et al. 2018). This “lack-of-fit problem,” originally developed in the context of a strong emphasis on internal validity and fidelity over external validity and contextual appropriateness in intervention research, has yielded EBPIs (and other HSRPs) that frequently demonstrate suboptimal fit with the individuals and settings for which they are intended (Lyon and Koerner 2016; Mohr et al. 2017a). Intervention-setting fit is a commonly-cited—but under-researched—determinant of implementation success (Aarons et al. 2012; Lyon et al. 2014a, b). These problems have contributed to a mental health care system that in large part does not accomplish consistent provision of evidence-based interventions (Bruns et al. 2015; Hoagwood et al. 2001).

## Digital Mental Health

Given this unmet need for high-quality mental health services for children and adolescents, researchers have devoted substantial attention to developing and evaluating digital mental health innovations to support access to youth mental health care in a cost-effective way (Hollis et al. 2017; Mohr et al. 2017a). Digital technologies developed for child and adolescent mental health include a large number of websites, apps, and technology-enabled services (TES, which have both digital and human components) that have been shown via randomized controlled trials to produce benefits similar to those of EBPIs, especially when paired with a human service component (Clarke et al. 2015; Mohr et al. 2017a; Seko et al. 2014). Although some digital mental health technologies are adaptations of more traditional EBPIs, this class of interventions need not be limited to existing protocols. Unfortunately, the rapid proliferation of such digital innovations to promote positive mental health has not coincided with intentional efforts to design technologies in a way that prioritizes the needs, workflows, and constraints of usual care settings (Mohr, et al. 2017a; Scholten and Granic 2019). Similar to EBPIs, digital innovations often are developed by teams that do not rely sufficiently on local expertise from intended implementation settings. Instead, they are developed by academic researchers and commercial teams, leading to a lack of fit between technologies and their intended users, which might include clinicians, service recipients, or the general public (Lyon et al. 2016c; Veinot et al. 2018). In addition to lack-of-fit, many digital health interventions fail to take advantage of the unique capabilities of digital technologies. In our experience, content development often occurs separately from technology development (i.e., designing the applications delivering the content), resulting in a poorly integrated experience. For example, it is common practice within digital health partnerships for researchers to develop the content and technology developers to design the app. This results in final products such as mobile websites with hierarchical menus and plentiful content, but without engaging or interactive elements that might increase the product’s appeal and overall effectiveness. Other times, digital tools are used to deliver essentially static interventions, without consideration of what else a mobile device or computer can do. Many models that guide the design of such interventions are based on an outdated understanding of the data available for use in the intervention and the limited interaction points with end users, which consequently fail to leverage the full potential of digital mental health. For instance, digital mental health designs sometimes default to a simple set of inputs and outputs that predate modern data sources (e.g., geo-positioning, accelerometer, heart rate, etc.). Thus, many available models inform designs that share these limitations (Riley et al. 2011). This contributes to poor uptake of technologies by clients, provider uncertainty regarding how to increase technology utilization, and technologies that do not fit naturally within existing care systems (Gilbody et al. 2015; Mohr et al. 2017a, b).

## Implementation Strategies

In support of these active efforts to advance EBPIs and digital interventions within mental health services research, an increasing number of implementation strategies (a third major category of HSRPs) have been developed to improve the adoption, high-fidelity use, and sustainment of interventions. Implementation strategies can be defined as methods or techniques used to enhance the adoption, implementation, and sustainment of a clinical program or practice (Curran et al. 2012; Proctor et al. 2013). Example strategies include initial training/educational meetings, post-training consultation, audit and feedback, identifying early adopters, changing liability laws, and training leadership, among many others (Powell et al. 2015). Optimizing the implementation of traditional and digital interventions has the potential to translate psychosocial intervention and digital mental health research into public health impact (Proctor et al. 2009). Unfortunately, the development and testing of implementation strategies has suffered from conceptual and terminological ambiguity, including a lack of consistency and clarity in reporting (Powell et al. 2015; Proctor et al. 2013). In response to these issues, the Expert Recommendations for Implementing Change (ERIC) project compiled a list of discrete implementation strategies and their definitions based on expert consensus (Powell et al. 2015). Similar compilations are being developed for common youth service sectors, such as schools (Cook et al. 2019; Lyon et al. 2019a). These efforts reflect a clear increase in attention to addressing determinants (i.e., barriers and facilitators) of implementation success to produce favorable implementation outcomes. This is critical given that over 600 unique, multilevel determinants of behavior change have been identified (Krause et al. 2014), including external policies and incentives, organizational culture, individual self-efficacy, and the relative advantage of interventions.

Despite growth in the field, work by implementation researchers and practitioners to identify effective implementation strategies has paid insufficient attention to innovation-level implementation determinants that are often “baked into” the design of both EBPIs (Lyon and Koerner 2016) and digital interventions (Mohr et al. 2017a). Although early implementation theories (e.g., Rogers 2003) emphasized aspects of innovations that lead them to be more or less likely to be adopted, there are few assessment tools or implementation strategies focused on understanding or addressing innovation-level determinants, such as design quality, complexity, and adaptability (Dopp et al. 2019b; Lewis et al. 2015; Lyon and Bruns 2019; Waltz et al. 2019). As a result, leading compilations of strategies (e.g., Powell et al. 2015) pay little attention to the intervention level. Furthermore, even less research has addressed the specific characteristics of implementation strategies that allow them to be more readily applied in real world service settings. A research agenda focused on the design of HSRPs, including EBPIs, digital interventions, and implementation strategies, has the potential to address these important gaps in the ultimate impact of youth mental health services.

## Over-Design and Under-Design in Contemporary HSRPs

As indicated above, HSRPs are often over-designed for research performance and under-designed for real world constraints. Youth mental health providers and other stakeholders (e.g., clients, administrators, EBPI purveyors) often encounter significant usability challenges with HSRPs, both in terms of the tasks involved (e.g., clinical techniques, goal setting, practice-specific supervision) and the packaging that structures the tasks (e.g., manuals, worksheets, length and modality of sessions). Some of these challenges could be addressed through improved attention to design during initial development. However, many HSRP design decisions are made in the context of high-resource conditions (e.g., a clinical trial at an academic medical center), which can inadvertently result in HSRPs that are optimized for such conditions and include numerous complex features that may not be necessary or feasible for users in other contexts (Lyon and Bruns 2019; Mohr et al. 2017a). This type of *over-design* is especially problematic given the historical emphasis on maintaining high fidelity during community-based applications of HSRPs. For EBPIs, this often involves requiring youth mental health service agencies to adopt wholesale a complex, expensive program without regard to its fit with their setting, clients, and available resources. A certain level of fidelity is likely necessary for any HSRP to produce its intended benefits, but intervention research has shown that “flexibility within fidelity” may actually produce better results than rigid adherence (Kendall et al. 2008; Park et al. 2018).

The inability of EBPIs to provide flexibility within fidelity is one example of how HSRPs are *under-designed* for typical service settings. Specialty mental health agencies and educational institutions are the two most common settings for youth mental health care (Center for Behavioral Health Statistics and Quality 2015), yet they are only represented in a small proportion of intervention development and testing research—generally, later-stage effectiveness research after the intervention has already been developed and demonstrated efficacy (Weisz et al. 2013). This is problematic because HSRPs are rarely designed to meet the needs of youth (e.g., frequent co-occurring problems), families (e.g., demographic diversity, including race/ethnicity and socioeconomic status), clinicians (e.g., large and diverse caseloads, often Master’s-level), implementation practitioners (e.g., work remotely with multiple organizations), and service organizations (e.g., emphasis on reimbursable activities) that represent typical delivery conditions (Weisz et al. 2013). Whether they are EBPIs, digital technologies, or implementation strategies, products that are over-designed in research settings lack sufficient local “run-time control” to allow flexible delivery in the moment to meet the complex, rapidly shifting demands of an active service environment (Chorpita and Daleiden 2014). Thus, when individuals are characterized as “resistant” or unsupportive of HSRPs, it may be that they are instead expressing legitimate concerns about how these interventions were not designed with their needs in mind.

HSRPs could be more impactful if they were more responsive to local constraints, suggesting that there may be benefits to moving into target settings—such as youth mental health agencies or schools—as soon as feasible in the design process. Deep structural changes are often needed in the redesign of HSRPs. That is, not simply modifying the appearance or user interface of products to make them contextually appropriate for different service systems. Instead, it may be necessary to challenge fundamental assumptions about how aspects of an EBPI, digital technology, or implementation strategy are structured (e.g., supporting clinician decision-making rather than providing a rigid, session-by-session manual) and organized within systems (e.g., recognition that front-line providers’ use of EBPIs is influenced by important organizational factors such as service structures). Although many of these issues are not unique to youth mental health, the future of youth mental health services requires innovative methods for assessing and improving the ease of use, utility, and contextual appropriateness of HSRPs.

## Human-Centered Design (HCD) Methods

The human-centered design (HCD) process reflects a set of methods that involve the development of products, technologies, and other artifacts for direct human use. HCD is characterized by a requirement that the human perspective is considered from the initial conception to the eventual design, and that the people who will use and/or be affected by the designed product (in this case, HSRPs) are involved in the process. The field and associated practices are also commonly referred to as user-centered design (UCD). However, a user-centric framing runs the risk of potentially overlooking the needs and priorities of non-user stakeholders in ways that can become problematic. For example, an EBPI protocol designed for use by school mental health providers will likely need to be designed with consideration of non-user (or non-primary user) stakeholders, including the client children, parents, and school administrators. Consequently, we use the term HCD. We also distinguish HCD from the more amorphous, but commonly used, concept of “design thinking.” Although “design thinking” has rapidly made its way into the popular lexicon, we find this conceptualization of design as a “mindset” to be too ambiguous and detached from specific methods, techniques, or behaviors to be a useful term.

**Fig. 1:**
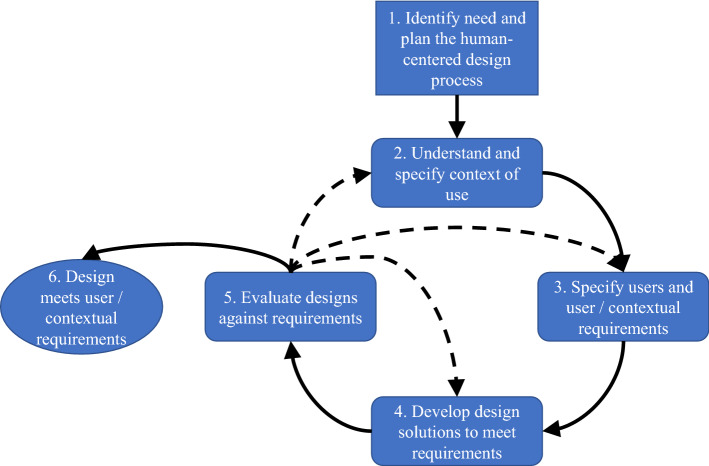
Generic, iterative human-centered design process, based on ISO 9241-210

HCD is typically applied to interactive technologies, and there is an international standard on its use in the design of interactive systems (ISO 1999). The International Standards Organization defines human-centered design as “an approach to interactive systems development that aims to make systems usable and useful by focusing on the users, their needs and requirements, and by applying human factors/ergonomics, usability knowledge, and techniques.” Many models describe the HCD process. While the details vary, each generally consists of investigating stakeholder needs and the context in which the product or service will be used, developing design ideas, prototyping one or more of those ideas at varying levels of “fidelity,” conducting initial evaluations with stakeholders, refining these prototypes and moving them toward fully functional prototypes, evaluating prototypes to see if they achieve their purpose and to understand unintended consequences, and eventually implementing and evaluating the results. The HCD process is iterative, and new barriers and results at any stage may prompt designers to revisit previous stages. Figure 1 displays a generic version of the HCD process, based on the International Standards Organization (ISO 1999).

A basic tenet of HCD is that its processes should result in parsimonious and accessible designs that are more usable for most potential stakeholders, regardless of their setting. Given observations that “the less change required, the more implementation may occur” (Aarons and Chaffin 2013), simplicity and other aspects of design quality that should result from redesign are expected to enhance scalability (i.e., the ability of an innovation to be used by greater numbers of people or systems) by reducing burden and resources. For instance, implementation strategies are sometimes needed to overcome low contextual fit for EBPIs. Although high-quality EBPI or digital technology design may never eliminate the need for implementation strategies, it has been suggested that better intervention design can lessen the need for implementation resources (von Thiele et al. 2019). Simultaneously, in acknowledgement of the potential for challenges with innovation generalizability (i.e., the applicability and maintenance of an innovation’s core assumptions) across settings and populations, a common HCD mantra is that “design for everyone is design for no one” (Design that Matters 2014). This suggests that, while generally parsimonious innovations are easier for anyone to implement, specific design decisions are likely to be most effective when they target specific users. Highly usable and scalable interfaces (for digital technologies, EBPIs, or implementation strategies) inevitably contain components that generalize across most (or all) groups as well as those that are tailored to particular contexts or user subgroups.

In addition, HCD shares some similarities with other approaches, such as community-based participatory research (Satcher 2005), especially surrounding the direct and meaningful incorporation of stakeholder perspectives (Oh 2018). However, HCD’s incorporation of stakeholders tends to be more targeted and episodic (e.g., collecting information from stakeholders/users throughout rather than always involving them directly in every phase) with final design decisions typically made by an independent design team. In this way, HCD is often a collection of methods for data collection and analysis (potentially including CBPR techniques) whereas CBPR typically articulates a more general reconceptualization of collaborators’ roles (e.g., shifting who is considered a researcher versus a participant).

Because HCD encompasses numerous methods for involving people in design (Maguire 2001), the process as a whole is difficult to assess for effectiveness. ISO 9241-210 claims that a human-centered design methodology enhances effectiveness and efficiency, improves human well-being, user satisfaction, accessibility and sustainability; and counteracts possible adverse effects of use on human health, safety and performance (ISO 1999). Individual methods within HCD have been shown to improve the usability of designed systems, and usable systems have been shown to have a number of positive outcomes, though a recent review of HCD in global health notes the difficulty in identifying clear, quantifiable outcomes for HCD as a whole (Bazzano et al. 2017). As described later (see Recommendations), there are important opportunities for research focused on HSRPs to also advance the general HCD literature.

## HCD in Health and Mental Health

HCD has begun to be recognized more specifically in the design of interactive health systems. For example, Johnson, Johnson, and Zhang (2005) analyzed HCD methods and provided a framework for how these techniques can be applied to the redesign of healthcare information systems. Many have also advocated for the use of HCD methods in consumer mobile health applications (e.g., McCurdie et al. 2012; Schnall et al. 2016). Additionally, researchers have applied HCD to innovate new systems to support the health of children and adolescents. This includes designing or adapting technologies to encourage children and youth to exercise more (Miller and Mynatt 2014; Toscos et al. 2006), manage their diabetes (Glasemann et al. 2010; Toscos et al. 2012), and engage in other healthy behaviors (Bisafar and Parker 2016). There have even been considerations for how personal health records could be tailored specifically to the needs of teenagers (Park et al. 2015). Together, these and other works illustrate how HCD can help identify the unique developmental, logistical, and social constraints and opportunities of children and adolescents and then design HSRPs accordingly.

Digital technologies are frequently recognized as holding promise for the application of explicit HCD methods, including in youth mental health (Bhattacharya et al. 2019; Matthews and Doherty 2011; Scholten and Granic 2019). Vacca (2017) engaged U.S. Latina adolescents in participatory workshops to explore needs and identify opportunities for them to access emotional support, thereby clarifying attitudes toward bicultural conflicts in emotional health. This work yielded a system for improving communication between Latina teens and their mothers (Vacca 2019). Work by Bruns et al. (2016) shows how HCD can improve healthcare for children even when they are not the user. They collected input from care coordinators and supervisors at multiple stages during the design of an electronic behavioral health information system for the wraparound care coordination model, helping to identify usability priorities and challenges. Design principles and methods have also been used to create measurement-feedback systems that collect and integrate information on the multiple perspectives involved in youth and family treatment (Bickman et al. 2012). Further, work by Lyon et al. (2016c) argues for the key role of design in adapting information technologies used in mental healthcare to novel settings, such as school-based mental health programs.

Although most applications of HCD methods in health continue to be in the context of digital systems, researchers are also beginning to consider the utility of this approach outside of the digital or health informatics space (Dearden et al. 2010; Dopp et al. 2019a; Roberts et al. 2016). For instance, using a process that emphasized stakeholder engagement and iterative prototyping, Hawkins et al. (2017) designed a peer-led public health intervention intended to prevent substance use among high school students. The intervention was “co-produced,” emphasizing the integration of empirical literature with stakeholder expertise and knowledge derived from a variety of qualitative methods (e.g., focus groups, observations). In mental health services, HCD has also begun to be applied to the complex patient-facing psychosocial interventions that dominate the evidence-based intervention landscape (Lyon and Bruns 2019). In service of this goal, Lyon and Koerner (2016) articulated a set of design goals for EBPIs: (1) learnability (i.e., provide opportunities to rapidly build facility in their use); (2) efficiency (i.e., minimize time, effort, and cost requirements); (3) memorability (i.e., easily remember and apply core components); (4) error reduction (i.e., prevent/recover from misapplications of content); (5) satisfaction or good reputation (i.e., acceptable and valuable compared to alternative products); (6) low cognitive load (i.e., simplified task structures); and (7) exploit natural constraints (i.e., explicitly address the static properties of a destination context). Collectively, these goals provide guidance for ensuring the implementability of EBPIs. Although they were designed to be specific to client-facing psychosocial interventions, these goals also apply to the design of implementation strategies.

Within the past decade, it has been increasingly recognized that implementation strategies also are complex psychosocial interventions (Proctor et al. 2013); albeit targeting different individuals to achieve different outcomes than EBPIs. Many implementation strategies are multi-faceted and multi-level (e.g., Aarons et al. 2017; Glisson and Schoenwald 2005; Kilbourne et al. 2007). For example, one strategy or “package” might include various components targeting practitioners (e.g., training, consultation), organizational leadership (e.g., facilitation of the change process), and service recipients (e.g., promotional materials). Thus, implementation strategies, like clinical interventions, also require explicit attention to design elements that can maximize their usability and effectiveness (Lyon et al. 2018). Information about the contextual fit of implementation strategies could support efforts to guide tailored selection and modification of strategies to meet local needs, as existing efforts have paid little attention to the characteristics of the strategies themselves (Baker et al. 2010; Powell et al. 2017; Wensing et al. 2009). “One-size-fits-all” implementation strategies that are overly complex, expensive, and difficult to use risk replicating, ironically, the very research-to-practice gap that implementation research seeks to overcome. An alternative approach is evident in the Interagency Collaborative Team model for implementing evidence-based treatments (Aarons et al. 2014; Hurlburt et al. 2014) whose developers explicitly co-designed the model with child welfare agency supervisors and leadership to promote effective collaboration across agencies. Moreover, recent research has suggested that HCD offers a useful complement to existing implementation strategies, few of which are able to attend to or modify intervention design elements to promote implementation success in novel settings (Dopp et al. 2019a, b).

When applied to HSRPs such as youth mental health interventions, technologies, and implementation strategies, HCD methods offer substantial opportunities to improve service quality through explicit inclusion of stakeholder perspectives and contextual needs during iterative development or redesign processes. Although attention to design is apparent in the success of some HSRPs, even these researchers typically have not explicitly embraced or referenced HCD—perhaps because design falls outside of traditional training in youth mental health services. More guidance on the best ways to incorporate HCD within the development and delivery of psychosocial interventions, technologies, and implementation strategies is clearly warranted. Attention to these issues is especially important in youth mental health given the complexity of most services (e.g., involvement of caregivers and other important adults, need to account for youths’ developmental levels) and the variety of service delivery settings (e.g., specialty mental health, schools, primary care, child welfare, juvenile justice).

## A Framework for HSRP Design

Although a wide range of frameworks leverage HCD principles and methods to improve the design of digital tools (e.g. Dwivedi et al. 2012; Humayoun et al. 2011; ISO 1999; Johnson et al. 2005; Mummah et al. 2016), including within the domains of youth (Druin 2002), intergenerational design (Walsh et al. 2013), and particularly of youth mental health (Scholten and Granic 2019), few frameworks have been developed to apply HCD principles to the development or redesign of EBPIs or implementation strategies. This is unfortunate, given the potential to improve the impact of a wide range of HSRPs by increasing local responsiveness and usability while avoiding both over- and under-design. Nevertheless, as noted above, some emerging work has begun to bridge this gap and leverage these methods to improve psychosocial mental health services (Bird et al. 2014; Lyon et al. 2019b; Mohr et al. 2017a). Among these advances, the Accelerated Creation To Sustainment (ACTS) framework (Mohr et al. 2017a) is one versatile approach, as it attends to the simultaneous redesign of digital technologies, psychosocial interventions, and implementation strategies. Concurrent consideration and improvement of these three types of HSRPs may create opportunities to be more impactful than considering any in isolation, as this acknowledges the increasingly frequent interdependence of these HSRPs in the mental health services landscape.

Based in HCD methods, Mohr et al. (2017a) developed the ACTS framework to support the design and evaluation of technology enabled services (i.e., mental healthcare services that have both a digital technology component and a human service component) as well as the implementation strategies intended to support them. Despite this targeted focus, the principles and steps of ACTS are broadly applicable to EBPIs and implementation strategies, regardless of whether they have a digital component. The ACTS model leverages stakeholder input to move the field toward more rapidly-developed and contextually-appropriate, yet simultaneously generalizable, innovations across three phases (Create, Trial, Sustain). Given evidence that careful design early in the development process will reduce the need for major downstream changes, an assumption of the ACTS framework is that, following an intensive early evaluation of user needs, redesign over subsequent iterations is likely to be increasingly minor. ACTS represents an example framework through which HCD methods can be leveraged to design or redesign EBPIs, technologies, and implementation strategies to advance youth mental health services by achieving three interconnected improvement goals for HSRPs: accessibility, effectiveness, and equity (see Table 2).

**Table 2:** Processes through which HCD can achieve HSRP accessibility, effectiveness, and equity

Goal	Proximal HSRP characteristics	Example tools and methods
Accessibility	Streamlined and scalable	Rapid prototyping Design probes
Effectiveness	Engaging and targeted	Identification of components Evaluation of mechanisms
Equity	Contextually appropriate and culturally relevant	User identification Contextual inquiry

### Accessibility

HCD carries considerable potential to improve the accessibility of high-quality youth mental health services by building streamlined HSRPs. Although it is tempting to build complex and comprehensive multi-layered digital or psychosocial products (Lyon and Bruns 2019; Nielsen and Loranger 2006), low-complexity innovations face fewer barriers to widespread adoption and sustainment, thus improving their accessibility (Aarons and Chaffin 2013; Rogers 2003; Torugsa and Arundel 2016). Within the ACTS framework (and HCD more generally), it is common to engage in rapid initial design as well as ongoing iterative development to ensure both parsimony and fit-to-purpose. Applied to HSRPs, an expectation of iterative development and “failing fast” relieves the often misplaced assumption that early attempts at EBPI, technology, or implementation strategy development should reflect a relatively final product. The ACTS *Create* phase focuses only on developing a “minimally viable product” for testing and rapid prototyping, in which design teams should be expected to fail often before arriving at an appropriate design solution. Rapid prototyping methods have strong evidence supporting their utility in improving the alignment of products with user needs and overall usability (Gordon and Bieman 1995), but have been applied only sparingly to youth mental health services. Prototyping is distinguished from more traditional pilot testing by its rapid and iterative nature, and its focus on challenging—versus confirming—core assumptions of the model (Lyon and Koerner 2016).

Although rapid prototyping methods can improve the accessibility of any HSRP, their application within digital technologies is most widespread. Creating accessible technologies for families requires attention to an even more diverse set of characteristics than designing for single users, including varying developmental stages and generational differences in technology use, necessitating careful selection of methods. For example, development of a behavioral intervention technology for both youth and caregiver users may be facilitated by *technology probes*, a strategy for introducing a new innovation into a complex everyday environment, such as a family system, to observe how it interacts with, and potentially changes, the people and setting. This approach also facilitates assessing the feasibility and robustness of the technology and its suitability for longer-term deployments (Hutchinson et al. 2003). Measurement-feedback systems (Bickman 2008) are a popular type of progress monitoring and decision support technology that facilitate the use of measurement-based care (Scott and Lewis 2015) in practice by automating data collection, summarization/synthesis, and display; the development of which could be facilitated by technology probes. Although a wide range of measurement-feedback systems have been developed, many of which have been specifically designed for youth and families, few have undergone a deliberate user-centered prototyping process (Lyon et al. 2016a, b, c). A key feature of some feedback systems is the ability to gather data on client and family functioning remotely (e.g., via a web portal) prior to treatment sessions, but differences across clients have been observed in the extent to which they routinely access these functions (Liu et al. 2019). In this scenario, researchers could introduce technology probes to examine how youth and their caregivers prepare for upcoming therapy sessions and when they interact with other technologies (e.g., smartphones, social media, or other software that is not the feedback system), potentially generating alternative strategies for system engagement and data collection. Finally, accessibility may also be enhanced by the extent to which target user groups are aware of the availability of products. Although dissemination, defined as targeted distribution of information to specific groups (Greenhalgh et al. 2004), is not a primary focus of HCD, better designed HSRPs are more likely to passively spread through service systems (e.g., creating “pull” for adoption via word of mouth; Rogers 2003) as a function of their compelling and engaging interfaces.

### Effectiveness

The effectiveness of HSRPs can also be enhanced via HCD methods that either improve product usability, enhance engagement, or increase precision by targeting specific HSRP mechanisms of action. Usability and user engagement are often key goals of HCD processes (Sutcliffe 2009), based on an assumption that well-designed innovations will encourage users to adopt and continue to use a project. When considering the effectiveness of healthcare products, HCD scholars have cautioned that researchers should move beyond considering clinical outcomes as the sole indicator of effectiveness. Indeed, early stage product development, or redesign, can most efficiently focus on more proximal variables—such as predictors of health benefits—prior to evaluating downstream effects on health status (Klasnja et al. 2011). Such attention to proximal variables can conserve resources (because these factors often emerge sooner and are less expensive to measure), allowing for more agile approaches to scientific discovery, optimizing interventions, and aggregating findings across studies (Klasnja et al. 2017). Mechanistic research relies on strong conceptual models, including theories that explain how HSRPs function (Lewis et al. 2018; Williams and Beidas 2019). The logic chain through which well-designed EBPIs, technologies, or implementation strategies have their impacts on users often includes improved engagement, usability, and implementation outcomes (e.g., adoption and high-fidelity use), as well as the mechanisms of action for the innovation itself.

The focus on proximal mechanisms in HCD is well aligned with a growing emphasis on the mechanisms through which mental health interventions (Kazdin 2007), digital technologies (Lyon et al. 2016b), and implementation strategies (Lewis et al. 2018) have their intended effects. The ACTS framework emphasizes effectiveness most explicitly in the *Trial* phase (Mohr et al. 2017a), the focal point of which is an optimization-effectiveness-implementation (OEI) trial that expands beyond well-established effectiveness-implementation trial designs (Curran et al. 2012) to optimize innovations and ensure that they are usable, effective, and implementable in their target setting. Through its inclusion of an optimization target (i.e., the extent to which an intervention is free of usability issues and demonstrates good fit-to-context), the OEI trial facilitates attention to key innovation-level mechanisms through which contextualized HSRP design can influence outcomes, including engagement and usability. To achieve this, optimization may involve end user testing, collection of real-world use data, or feedback interviews with stakeholders. Related, HCD also has the potential to inform and advance the goal of effective HSRPs in youth mental health by facilitating a more nuanced understanding of how the components of specific EBPIs, technologies, and implementation strategies have their effects; provided there is a good theoretical model that articulates how the HSRP functions. For instance, one study seeking to iteratively develop and optimize a brief post-training consultation implementation strategy for school-based clinicians (Lyon et al. 2018) identified three key mechanisms for its consultation model: collaboration, responsiveness, and accountability. Iterative design and testing (via cognitive walkthroughs [Mahatody et al. 2010] and small-scale microtrials [Howe et al. 2010; Leijten et al. 2015]) were employed to address the extent to which the strategy was engaging, usable, and influenced its target mechanisms. Through multiple iterations, the strategy maximized these outcomes in advance of a larger-scale trial focused on implementation outcomes.

### Equity

Equity in mental health service access and service outcomes has received increasing international attention (Goddard and Smith 2001; Green et al. 2013; Patel et al. 2010; Vasiliadis et al. 2005). Equity is defined as the absence of systematic disparities between groups with different levels of underlying social advantage/disadvantage (Braveman and Gruskin 2003). Even when interventions, technologies, or implementation strategies may be effective overall, they can still inadvertently increase inequities if their effects differ between different groups of users (e.g., Liu et al. 2019; Veinot et al. 2018). HCD can support equity goals in youth mental health services by improving the contextual appropriateness and cultural responsiveness of HSRPs. Although appropriateness and responsiveness are not the only pathways to achieving service equity [e.g., equity can also be enhanced by improving service access (see above), among a variety of other multilevel targets (Kilbourne et al. 2006)], they provide promising targets to reduce healthcare disparities.

A core principle of HCD is that stakeholders are in the best position to communicate their needs to design teams. This is often achieved via explicit identification of user characteristics (Kujala and Kauppinen 2004) as well as participatory design processes (e.g. Vacca 2017,). Inequities in healthcare often occur as a result of an inadequate understanding or incorporation of user needs since developers who lack understanding of end users and other stakeholders are likely to base designs on their own needs (Kujala and Kauppinen 2004; Kujala and Mäntylä 2000). For these reasons, HCD may be especially applicable to developing interventions for underserved populations (Altman et al. 2018). The incorporation of user perspectives to promote equity is critical across all phases of the ACTS framework, ranging from the *Create* phase (e.g., identify users and their needs) through the *Trial* phase (e.g., evaluate the extent to which the HSRP meets core user needs across groups) and to the *Sustainment* phase (e.g., use passive or other low-burden, pragmatic data to determine whether disparities emerge in use patterns over time). Nevertheless, it is worth noting that user needs are not always exactly as stakeholders describe them. As Giacomin (2014) puts it, design methods support “…obtaining an understanding of their needs, desires and experiences which often transcends that which the people themselves actually knew and realized.” Approaches that integrate data from multiple sources of information (e.g., interviews/focus groups, observation, object-based techniques) can help designers and researchers gain such an understanding.

Early contextual inquiry can help avoid equity-related pitfalls by surfacing critical information about the destination setting and can serve as the foundation for local intervention redesign (Lyon and Bruns 2019). In addition, newer methods, such as remote approaches (e.g., that allow for geographically distant and asynchronous co-design among intergenerational collaborators; Walsh et al. 2012), conducting participatory design in virtual game environments familiar to youth (Walsh et al. 2015), and hosting design groups in community spaces, such as local libraries (Yip and Lee 2018) can engage families from underrepresented populations, increasing their voice in the design process. Furthermore, not all interventions are equivalent with regard to their ability to be responsive to context without violating their core assumptions or structures. For instance, although EBPI manuals are typically constructed with the assumption of consistent sequencing and duration of context, newer modularized interventions (e.g., Modular Approach to Therapy with Children [Chorpita et al. 2017; Weisz 2012]; CETA [Murray et al. 2014]) provide opportunities for greater flexibility in content for youth with co-occurring clinical problems.

Increased flexibility can facilitate the design of EBPIs that more deliberately balance research evidence and local evidence and allow for the incorporation of novel, locally-relevant content that meets the needs of specific settings or cultural groups (Lyon et al. 2014a, b). In this scenario, early structured contextual inquiry (e.g., observations, ethnography, workflow analysis, interviews, etc.; Holtzblatt and Beyer 2017; Holtzblatt et al. 2004) can yield an initial design document, which provides details about local constraints and anticipated product specifications (Vredenburg et al. 2002) and can drive the design or redesign of EBPIs, as well as other HSRPs.

## Special Considerations for Designing HSRPs for Youth Mental Health Services

HCD carries significant opportunities to improve the reach of EBPIs, digital technologies, and implementation strategies. A number of special considerations apply, however, when considering how to leverage HSRPs to advance the future of youth mental health services. These include the involvement of multiple core users, spanning ecological contexts, and the need for developmentally-appropriate evaluation techniques. Below, we detail each of these considerations, as well as methods that may help to address them.

First, youth mental health services invariably involve more individuals and collateral contacts (e.g., caregivers, teachers, other family members) than is typical in adult services. As a result, HSRPs that are intended to improve youth mental health services tend to have a wider array of identifiable primary or secondary users. Primary users are the target group for a product whose needs are prioritized in the design or redesign process, whereas secondary users are those whose needs can be accommodated as long as they do not compromise a product’s ability to meet the primary users’ needs (Cooper et al. 2007). For youth mental health services, primary users often include the youth themselves, their caregiver(s), and service provider(s). Secondary users may include teachers (or, in the case of students involved in special education, other support staff), family members, or administrators who make adoption decisions about HSRPs within service agencies. This array of potentially critical stakeholder perspectives requires careful attention to user identification, which can be optimized by incorporating a systematic user identification process (e.g., Kujala and Kauppinen 2004). This may be coupled with participatory HSRP design processes that incorporate design partnerships between adults and children (e.g., Druin 1999; Yip et al. 2017), especially in cases (such as parent training EBPIs) that rely on effective interactions for therapeutic effects.

Second, because youth tend to spend time in multiple relevant ecological contexts (e.g., home, school, peers, after school, specialty mental health, primary care), new HSRP innovations may be designed to transcend any specific setting to promote accessibility, effectiveness, and equity. Cloud-based digital products may most easily span settings (e.g., creating mental health promoting games [Fleming et al. 2017] that can be prescribed, used, and supported in different contexts), but EBPIs and implementation strategies can also be designed for cross-context use. For example, designing for cross-context use may include developing implementation strategies (e.g., building coalitions, modeling and simulating change; Powell et al. 2015) that explicitly span the boundaries of multiple settings to promote adoption and sustainment of new programs or intentionally developing different versions of strategies that target key users across contexts.

Finally, although user testing is a mainstay of HCD, the applicability of many industry-standard testing techniques to youth is less well established than with adult populations (Hourcade 2008; Markopoulos and Bekker 2003). While this is unlikely to be problematic for the dedicated caregiver-facing aspects of existing EBPIs or digital technologies (e.g., caregiver psychoeducation; caregiver portals in an electronic health record), children and adolescents differ from adults on key variables that may impact a usability assessment process, including verbal ability, attention span, motivation, sensitivity to unfamiliar environments, ability to provide valid self-report, abstract cognition, and fund of knowledge, among others (Markopoulos and Bekker 2003). Information gathering approaches that are sensitive to the needs and abilities of youth often involve gathering data from multiple youth simultaneously and might include peer tutoring with interviews, in which one child or adolescent first learns about a system and then becomes an instructor for the other during a usability evaluation (Edwards and Benedyk 2007), or group testing (Kantosalo and Riihiaho 2019). In addition, careful consideration of the power dynamic between adults and youth is a key aspect of successful design with children (Druin 1999). This may be achieved through intentional efforts to build a positive relationship throughout the design process. Adolescents in particular have unique needs in terms of recruitment and consent, power imbalances, and need for adaptation of standard HCD methods (Poole and Peyton 2013).

## Recommendations and Conclusion

The ability of HSRPs to shift public mental health outcomes remains elusive due, in part, to a persistent disconnect between the contexts where those products were developed and the settings in which children and adolescents receive care. HCD “knows how” to bring products in line with the needs of stakeholders, but principles and methods from HCD are underutilized relative to their potential to impact HSRPs in youth mental health, resulting in persistent over- and under-design. Future research and practice can help to close that gap and improve the acceptability, effectiveness, and equity of EBPIs, digital technologies, and implementation strategies. Below we offer some concluding recommendations for further integrating HCD and youth mental health services.

### Identify the Aspects of HCD that are Most Useful for Improving HSRPs in Youth Mental Health

HCD contains a wide variety of methods that are likely applicable in healthcare (Dopp et al. 2019a). Many, or perhaps even most, of these can be leveraged to improve the design of EBPIs, digital technologies, and implementation strategies, but additional data collection is needed to determine which methods have the greatest utility for identifying usability problems, driving redesign decisions, and improving implementation and service outcomes. As described above, many methods are untested with the children and adolescents who are core users of many HSRPs. From the array of HCD methods available, it is also important to select those that are most appropriate for use with EBPIs and implementation strategies. Otherwise, adaptation of those methods may be indicated (see below).

### Develop or Refine HCD Methods for Non-digital Products (i.e., EBPIs and Implementation Strategies)

Although most HCD methods are presumably applicable to digital technologies in youth mental health, psychosocial innovations (i.e., EBPIs and implementation strategies) may require more adaptation of these methods to maximize their potential (Lyon, Koerner, and Chung, under review). EBPIs and implementation strategies are particularly complex and often rely more on socially-mediated interactions among users (e.g., clinician and client; intervention purveyor and clinician) than on highly-structured visual interfaces. Some direction may be taken from the literature on “service design” (e.g., Freire and Sangiorgi 2010; Zomerdijk and Voss 2010), but because this is still an emerging field, service design methods tend to be less well developed, empirically rigorous, or reproducible than other domains within HCD.

### Evaluate Proximal Mechanisms for HCD Methods

As indicated above, health services and implementation research are increasingly focused on identifying and testing putative mechanisms of action through which interventions have their desired effects. Some mechanisms, most notably usability, have been identified for HCD, but because few examples exist of head-to-head comparative trials of the implementability and impact of innovations that have been developed with and without appropriate HCD methods, these have not been tested via the mediational models that are ideally indicated (Kazdin 2007). Randomized trials of HSPRs to HCD-driven redesign that track the impact on usability and outcomes are indicated. Beyond usability, additional potential mechanisms through which HCD processes influence behavioral implementation outcomes (e.g., adoption, sustainment) and client outcomes (e.g., symptom improvement) may include many perceptual implementation outcomes (e.g., acceptability to users, cultural relevance, developmental appropriateness, applicability to context) as well as factors such as behavioral intentions to use a product (Moullin et al. 2018).

### Ensure that HCD Research on HSRPs is “Backward Compatible”

Beyond the contributions of HCD to HSRPs, applications of HCD in mental health may also provide opportunities to generate novel findings that are “backward compatible”—meaning they can help to advance the HCD literature. As noted earlier, although an extensive literature has linked HCD processes and well-designed products to precursors of adoption and use (such as adoption intentions), very little research has examined the extent to which co-produced (with stakeholders) or better-designed products actually result in improved implementation or health service outcomes (Bombard et al. 2018). A recent systematic review of HCD in healthcare (Altman et al. 2018) identified that only four studies have explicitly compared redesigned interventions to original interventions, none of which were large-scale randomized trials and most of which were heavily focused on digital innovations. With funding from the National Institute of Mental Health, work is underway which will explicitly evaluate the impact of HSRP redesign on usability, implementation outcomes, and client outcomes by comparing original EBPIs and implementation strategies to redesigned versions (e.g., Lyon et al. 2019b), but additional research is sorely needed.

### Look to Where Necessity has Spawned Invention (e.g., Globally) in Methods and Design

Designers of HSRPs should be on the lookout for persistent workarounds and natural design solutions that emerge through everyday use in real-world contexts. For instance, if implementation practitioners commonly omit or revise components of a complex implementation strategy, then those components should be closely examined for redesign or removal. In a similar vein, local constraints in global mental health have given rise to a variety of innovative EBPI design solutions such as simplified intervention design for lay service providers (Rahman 2007; Rahman et al. 2008) or task shifting techniques that maximize the efficiency with which available expertise is distributed (Patel 2009).

### Develop New Roles and Collaborations to Support the Alignment of HCD and Implementation

Finally, given that no single individual, entity, or role is likely to be able to take full responsibility for the design or redesign of HSRPs, there is considerable utility in establishing new partnerships to advance the objectives detailed above. Indeed, effective teaming processes for HCD and implementation professionals have been identified as a particularly critical infrastructure to support the effective use of HCD techniques (Dopp et al. 2019b). At one level, developers of digital technologies, EBPIs, and implementation strategies may be best positioned to engage in HCD activities—such as usability testing—during initial development or in preparation for large-scale implementation efforts. However, this is best done in partnership with other stakeholders who can also take on meaningful responsibilities. For instance, Lyon and Bruns (2019) suggested that purchasers of care (e.g., state Medicaid officials and insurers) might collaborate with health care organizations to convene stakeholders (possibly including, but not limited to, developers) in collaborative redesign teams when introducing EBPIs or other HSRPs into new contexts. Additional groups that have traditionally been highly patient-centered – such as patient advocacy organizations, patient advisory boards, and even federal research sponsors (e.g., the Patient-Centered Outcomes Research Institute [PCORI; Selby and Lipstein 2014])—might help to lead these collaborative efforts. Overall, it is likely that widespread adoption of HCD methods will, itself, be facilitated by explicit use of a subset of the wide array of implementation frameworks and strategies that have been developed (Nilsen 2015; Powell et al. 2015). Although these approaches have typically been applied to human service innovations (e.g., interventions or other HSRPs), recent work has articulated the relevance of such frameworks and strategies to a broader set of innovations such as quantitative research methods and analytic techniques (King et al. 2019).

In sum, human-centered redesign of HSRPs provides an innovative and timely pathway for improving the public health impact of our best innovations by restructuring—and sometimes reimagining—them to enhance their feasibility and practicality for use in public sector youth service contexts. The HCD literature consistently reinforces the perspective that engaging in systematic, approaches to design is less expensive early in development and likely to yield cost-savings in the long run (Vredenburg et al. 2002), and the costs of engaging in effective HCD are likely to pale in comparison to the costs of failed implementations. While some limited work has sought to articulate the potential costs of related “co-production” activities (Oliver et al. 2019) the specific costs of HCD when applied outside of digital technologies (i.e., to EBPIs and implementation strategies) is a critical avenue for future research.

In modern mental health services, interventions, technologies, and implementation strategies increasingly interact. Framing our discussion above using the ACTS model acknowledges that the simultaneous consideration of these components of service improvement creates opportunities to be more impactful than considering any one in isolation, but redesign of any single component is still likely to improve its unique impact and opportunity for large-scale use. We invite all researchers and practitioners to consider how HSRPs can better fit the needs of the stakeholders and contexts with which they are applied as we all design the future, in real time, of youth mental health services.
